# Identifying Molecular Probes for Fluorescence-Guided Surgery in Neuroblastoma: A Systematic Review

**DOI:** 10.3390/children12050550

**Published:** 2025-04-24

**Authors:** Megan Hennessy, Jonathan J. Neville, Laura Privitera, Adam Sedgwick, John Anderson, Stefano Giuliani

**Affiliations:** 1 Cancer Section, Developmental Biology and Cancer Programme, UCL Great Ormond Street Institute of Child Health, London WC1N 1EH, UK; megan.hennessy1@nhs.net (M.H.); jonathan.neville@nhs.net (J.J.N.); l.privitera@ucl.ac.uk (L.P.); j.anderson@ucl.ac.uk (J.A.); 2 Royal Free Hospital NHS Foundation Trust, London NW3 2QG, UK; 3 Department of Specialist Neonatal and Paediatric Surgery, Great Ormond Street Hospital for Children NHS Trust, London WC1N 3JH, UK; 4 Department of Chemistry, King’s College London, London SE1 1DB, UK; adam.sedgwick@kcl.ac.uk; 5 Wellcome/EPSRC Centre for Interventional and Surgical Sciences, University College London, London W1W 7TS, UK

**Keywords:** paediatric surgery, neuroblastoma, fluorescence-guided surgery, image-guided surgery, systematic review

## Abstract

**Background/Objectives**: Targeted and non-targeted fluorescent molecular probes (FMPs) can be used intra-operatively to visualise tumour tissue. Multiple probes have been clinically approved for fluorescence-guided surgery (FGS) in adult oncology, and the translation of these technologies to paediatric neuroblastoma may provide novel strategies for optimising tumour resection whilst minimising morbidity. We aimed to identify clinically approved FMPs with potential utility for FGS in neuroblastoma. **Methods**: A systematic review of the literature was performed in accordance with the PRISMA guidelines (PROSPERO CRD42024541623). PubMed and Web of Science databases were searched to identify studies investigating clinically approved FGS probes and/or their targets in the context of neuroblastoma. Pre-clinical and clinical studies looking at human neuroblastoma were included. The primary outcomes were that the FGS probe was tested in patients with neuroblastoma, the probe selectively accumulated in neuroblastoma tissue, or that the target of the probe was selectively over-expressed in neuroblastoma tissue. **Results**: Forty-two studies were included. Four were clinical studies, and the remainder were pre-clinical studies using human neuroblastoma cell lines, human tumour tissue, or xenograft models using human neuroblastoma cells. The only FMP clinically evaluated in neuroblastoma is indocyanine green (ICG). FMP targets that have been investigated in neuroblastoma include poly-ADP ribose polymerase (PARP) (targeted by PARPiFL), endothelial growth factor receptor (EGFR) (targeted by Panitumumab-IRDye800CW, Cetuximab-IRDye800CW, Nimotuzumab-IRDye800CW and QRHKPRE-Cy5), vascular endothelial growth factor receptor (VEGFR) (targeted by Bevacizumab IRDye800CW), and proteases such as cathepsins and matrix metalloproteinases that activate the fluorescent signal of FMPs, such as LUM015 and AVB-620. Of the clinical studies included, all were found to have a high risk of bias. **Conclusions**: ICG is the only clinically approved fluorescent dye currently used for FGS in neuroblastoma; however, studies suggest that its ability to recognise neuroblastoma tissue is inconsistent. There are several clinically approved FMPs, or FMPs in clinical trials, that are used in adult oncology surgery that have targets expressed in neuroblastoma. Further research should validate these probes in neuroblastoma to enable their rapid translation into clinical practice.

## 1. Introduction

Neuroblastoma is the most common extracranial solid tumour in children and is responsible for 10–15% of paediatric oncology mortality [1]. The majority of patients have high-risk disease, which can be refractory to intensive treatment, resulting in an overall 5-year survival rate of less than 50% [2]. The potential for metastasis is high, and around two-thirds of patients present with tumours that have already spread at the time of diagnosis [3].

Surgery remains a critical component of the multimodal treatment of neuroblastoma. Complete macroscopic resection of the tumour is associated with higher overall and event-free survival, and a lower incidence of local disease progression [4]. However, neuroblastoma resection is challenging as the tumour is often adherent to critical anatomical structures. As such, neuroblastoma resection is associated with a significant risk of morbidity. In addition, following neoadjuvant chemotherapy, it is difficult to discriminate between viable and non-viable tumour tissue. Surgeons must therefore balance their attempts to achieve a complete macroscopic resection of viable tumours whilst minimising morbidity. One potential tool to assist surgeons is fluorescence-guided surgery (FGS). The use of fluorescent molecular probes (FMP) to label tumour tissue facilitates margin identification intra-operatively, potentially enabling complete resection whilst minimising damage to the surrounding unlabelled structures. FGS may also be useful in discriminating between viable and non-viable tumour tissue and hence assist in intra-operative decision making.

Many clinically approved FMPs are used routinely in adult patients with cancer. These include non-targeted probes such as indocyanine green (ICG), methylene blue, and fluorescein, which work by binding to plasma components and have been shown to passively accumulate in tumours. This phenomenon is best explained by the enhanced permeability and retention (EPR) effect, whereby the presence of defective endothelial cells and wide fenestrations in the blood vessels of tumour tissue permit the uptake of FMPs. Once taken into the tumour microenvironment, they are retained due to properties such as their molecular size and polarity, as opposed to targeting mechanisms such as ligand–receptor interactions [5]. This is, however, not tumour-specific and subject to false positives and negatives depending on the extent of tumour blood flow and other factors increasing the presence of vascular mediators in tissue. Targeted probes such as PARPiFL (targeting poly-ADP ribose polymerase (PARP)) are also available, which work by binding selectively to targets that are overexpressed in cancer cells and are therefore more specific to tumour tissue. There are also a class of activatable FMPs, such as LUM015, which is cleaved by cathepsin proteases in the tumour microenvironment into an optically active fragment.

Clinically approved probes have been validated in patients, showing efficacy as FGS agents with tolerable side-effect profiles. While the use of FGS in paediatric oncology remains in the developmental stages of innovation, it may be the case that FMPs validated in adult patients with cancer show similar efficacy in children with neuroblastoma [6]. For non-targeted probes, this would mean probe accumulation in neuroblastoma tissues relative to normal tissues, whereas targeted probes require the specific expression of a target by neuroblastoma cells or the over-expression of a target by neuroblastoma cells relative to non-tumour tissue. The identification of clinically approved FMPs which meet these criteria would enable a more straightforward translation to paediatric oncology surgery.

In this systematic review, we aim to identify clinically approved probes that have potential utility for FGS in children with neuroblastoma, with the hope of focusing future research and accelerating the translation of new FMPs into clinical practice.

## 2. Materials and Methods

A systematic review of the literature and a meta-analysis were performed according to the Preferred Reporting Items for Systematic Reviews and Meta-Analysis (PRISMA) guidelines [7]. The study protocol was specified in advance and pre-registered in the international prospective register of systematic reviews (PROSPERO) [CRD42024541623].

A list of FMPs that are currently approved for use in FGS in cancer, or that are undergoing testing in clinical trials, was compiled from a literature search on PubMed and a search of clinicaltrials.gov. The included probes and relevant clinical trial numbers are outlined in Table 1. Using these probes, an electronic database search was performed of MEDLINE via PubMed and Web of Science from the inception of the database until the 10 February 2024 (Supplementary Materials S1). The search was subsequently re-run on 25 November 2025 in order to identify studies published between the search dates. Reference lists were also screened.

Studies that were eligible for inclusion were those that included children (≤18 years of age) diagnosed with neuroblastoma or human neuroblastoma tumour tissue or cells ex vivo and investigated the use of clinically approved FGS probes or their targets. Review articles, case reports, animal studies, and studies not published in English were excluded. Two reviewers (M.H. and J.J.N.) independently screened the titles and abstracts of each study identified from the literature search using the abstract decision tree found in Supplementary Materials S2. Articles failing to meet the inclusion criteria and duplicates were excluded. The full text of the selected articles was assessed against the predefined inclusion criteria.

Data were then extracted by M.H. on a predefined Microsoft Excel spreadsheet and validated by J.J.N. independently. The extracted data included the following:

1. Study characteristics: authors, year, country of study, study design, and FMP or target investigated.
2. Patient demographics: patient cohort size, age range, sex, number of tumours, tumour location, and tumour stage and grade.
3. Pre-clinical study features: sample type (cell lines, tissue samples, xenograft models using human neuroblastoma cells), sample size, study aim, methods, results, and conclusion.

The primary study outcomes were that the FGS probe was tested in patients with neuroblastoma, the probe selectively accumulated in neuroblastoma tissue, or that the target of the probe was selectively over-expressed in neuroblastoma tissue.

An assessment of the risk of bias in clinical studies was undertaken using the Risk of Bias In Non-Randomized Studies of Interventions (ROBINS-I) tool for non-randomised intervention studies. No studies were excluded based on the risk of bias assessment. Due to the heterogeneity of the included studies and the under-reporting of patient demographic information and outcome, a formal statistical analysis could not be performed. Hence, the following review aims to provide a descriptive analysis of the included studies.

## 3. Results

**Figure 1:**
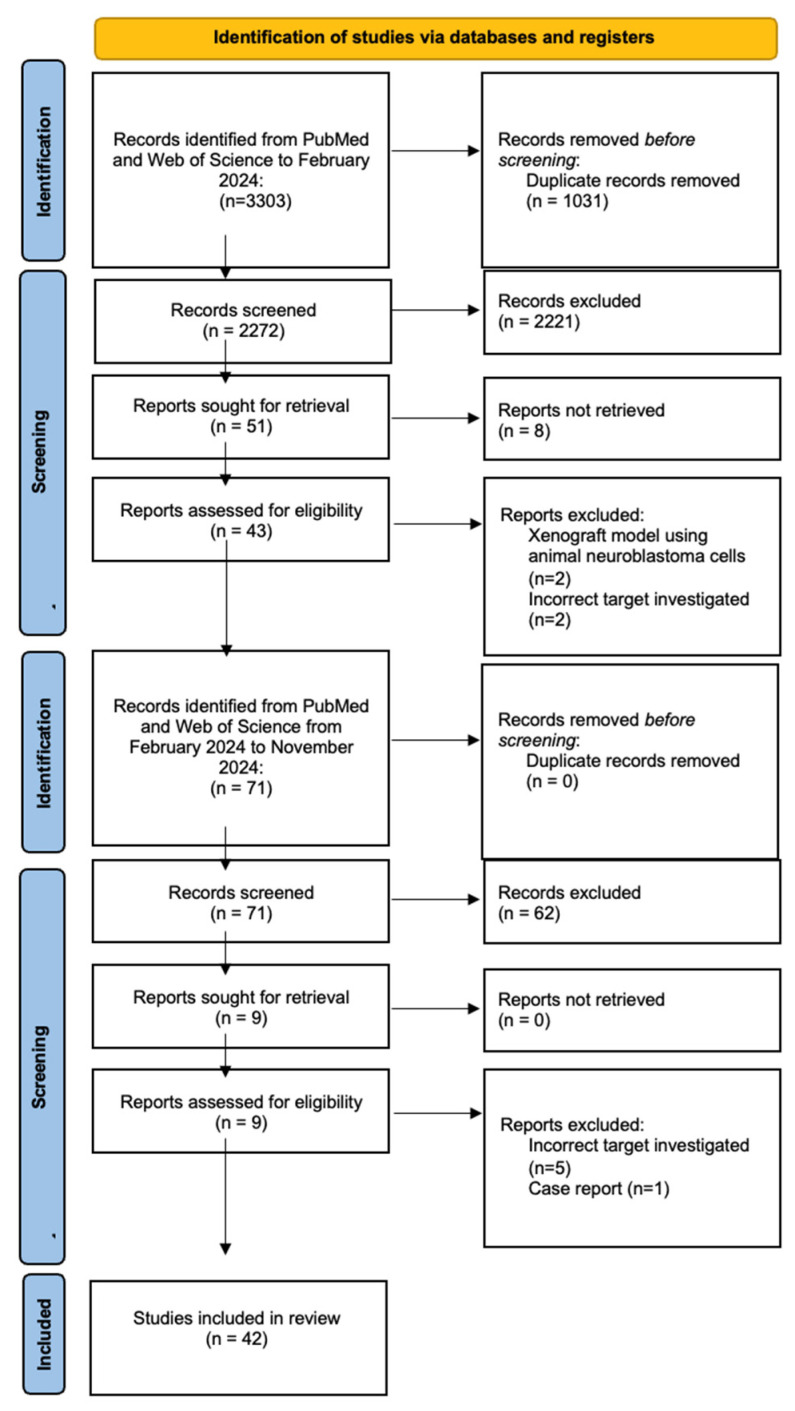
Flow diagram of the study identification and selection process, following the PRISMA guidelines [7].

A total of 3374 studies were identified from the database search (Figure 1). Duplicate results and articles not published in English were excluded, and 2343 studies underwent title and abstract screening. Of these, 52 underwent a full-text review and 42 studies were selected for inclusion [8,9,10,11,12,13,14,15,16,17,18,19,20,21,22,23,24,25,26,27,28,29,30,31,32,33,34,35,36,37,38,39,40,41,42,43,44,45,46,47,48,49]. No studies were identified following the screening of reference lists.

Of the 42 included studies, four (10%) were clinical and 38 (90%) were pre-clinical (Table 2). Of the clinical studies, three were phase 1 clinical trials and the other was a retrospective cohort study. The pre-clinical studies used a range of sample types: six included xenograft mouse models derived from human cell lines, 14 used patient-derived tissue samples, and 30 used neuroblastoma cell lines. Two screened cancer genetics databases. Fifteen studies used more than one type of sample [11,17,22,23,25,27,29,31,32,33,38,43,44,45,46].

### 3.1. Clinically Investigated FMPs or Targets 

There were three clinical studies of approved FMPs and one clinical study of an FGS target in phase 1–2 trials applied to neuroblastoma, the cohorts of which are described in Table 3 [8,9,20,39].

#### 3.1.1. Studies Involving Approved Probes

##### ICG

Given its approval status and established use in FGS, the only FMP studied clinically was ICG. This is an untargeted dye which is preferentially taken up in tumour tissue through the EPR effect. This involves the binding of ICG to intravascular plasma proteins, which can leak into tumour tissue via porous vasculature and be retained in the tumour environment due to impaired lymphatic drainage.

In a retrospective review of six neuroblastoma tumours that underwent an FGS tumour resection between 2019 and 2020, ICG was found to have high sensitivity and specificity for malignant tissue intra-operatively, with five of the studied tumours found to fluoresce [8]. No neuroblastoma tumours were identified using ICG fluorescence that were not already identified using the standard-of-care white light.

However, in a subsequent study by the same group aiming to use ICG to localise pulmonary metastases of paediatric solid tumours, including one neuroblastoma patient with 17 nodules, ICG failed to localise any of the nodules that were later identified intra-operatively via direct visual inspection or palpation without a fluorescent signal [9].

In a recent open-label single-centre phase 1 clinical trial, ICG was administered at the induction of anaesthesia to further investigate the utility of ICG in showing the margins between normal tissue and tumour [20]. Whilst two thoracic neuroblastoma were found to fluoresce, with no residual tumour seen on post-operative MRI, the suprarenal neuroblastoma did not, demonstrating anatomical variability in ICG’s utility.

Beyond its role in intra-operative imaging, ICG has also been investigated in a pre-clinical study for its potential application in photodynamic therapy in neuroblastoma cell lines. Ak et al. explored whether the use of different laser parameters (50 J/cm^2^ and 100 J/cm^2^), combined with different concentrations of ICG (25 ug/mL and 50 ug/mL), has cytotoxic and anti-proliferative effects on neuroblastoma cell lines [10]. The findings from the study showed decreased cell proliferation in a dose-dependent manner compared with controls, with cytotoxic effects on neuroblastoma cell lines at the 50 ug/mL dose. There was no difference between treatment groups with laser treatment alone; however, photodynamic therapy using lasers combined with ICG showed a significant reduction in cell viability at both concentrations of ICG, with a greater effect at the higher concentration.

#### 3.1.2. Studies Involving Probes/Targets in Phase 1–2 Trials

##### PARP

Poly (ADP-ribose) polymerase (PARP) is a family of proteins involved in the detection and repair of DNA strand breaks. PARP inhibitors (PARPi) such as Olaparib have been widely investigated for use in oncology. The FMP, PARPiFL, was designed to target PARP and is currently undergoing human trials. Although PARPiFL has not been directly studied in neuroblastoma, the role of PARP as a therapeutic target for neuroblastoma has been investigated.

A phase 1, open-label, multi-centre clinical trial investigated the efficacy of Olaparib in paediatric patients with refractory solid tumours [39]. Of the six neuroblastoma patients included, one showed a partial tumour response to Olaparib, and the drug was shown to be well tolerated.

PARP expression in neuroblastoma, as well as the therapeutic potential of PARPi, was also investigated pre-clinically in neuroblastoma-derived tissues and cells, with nine identified [15,16,22,24,26,28,32,38,48].

Makvandi et al. analysed RNA sequencing data from 126 high-risk primary neuroblastoma cases and found that PARP-1 expression was significantly elevated in neuroblastoma compared to 31 types of healthy tissue, including adipose, lung, and oesophageal tissues [26]. PARP-1 expression was also found to be associated with decreased survival in high-risk patients, though the same correlation was not observed in low-risk cases.

Similarly, Colicchia et al.’s study, a gene expression analysis of 498 human neuroblastoma samples, found PARP-1 expression to be higher at advanced stages of neuroblastoma and MYCN-amplified tumours [15]. In a later study, it was shown that MYCN expression increased sensitivity to PARP inhibition, and MYCN-expressing cells demonstrated significantly reduced cell survival following PARP inhibition compared with non-MYCN-expressing cells. Switching off MYCN was shown to slightly reduce the expression of the PARP-1 protein. In a xenograft model of mice bearing MYCN-expressing tumours, treatment with Olaparib was shown to significantly prolong survival compared with untreated controls [24].

A 2023 screening of the Genomics of Drug Sensitivity in Cancer database revealed that PARPi demonstrated the most significant efficacy against paediatric cancer cell lines [22]. Several studies investigated use of the PARPi Olaparib in neuroblastoma, often in combination with other chemotherapeutic agents [16,28,32]. In neuroblastoma cell lines, Olaparib was shown to enhance the toxicity of the cytotoxic agents with which it was paired. When its effect was studied in xenograft models, it was shown that some neuroblastoma cell lines were sensitive to Olaparib, particularly those with mutations in genes associated with the DNA damage response (DDR) pathway, of which the ATM gene is thought to be the master regulator. Whilst the attenuation of tumour cell growth was observed in DDR-defective neuroblastoma cell lines treated with Olaparib, this effect was not seen in ATM-competent ones, suggesting that pre-existing defects in DNA damage repair pathways increases tumour sensitivity to PARPi [38].

### 3.2. Pre-Clinically Investigated FMPs or Targets

#### 3.2.1. Studies Involving Approved Probes or Their Targets

##### 5-Aminolevulinic Acid (5-ALA)

5-ALA is an approved pro-agent for FGS in malignant glioma. It is a natural biochemical precursor of haem that is metabolised to fluorescent porphyrins, such as PPIX, and exogenous administration of the probe allows for the accumulation of these compounds in target tissue.

One study assessed the susceptibility of human neuroblastoma cell lines to photodynamic therapy with 5-ALA [42]. Whilst 5-ALA alone did not show cytotoxicity in any cell line, irradiation of the treated cells resulted in phototoxicity, with 35% of cells expressing apoptotic markers after 24 h.

##### LUM015 and Cathepsins

LUM015 (or LUMISIGHT or pegulicianine) is an activatable FMP that produces a fluorescent signal after its peptide chain is cleaved by cathepsins. It works preferentially in malignant tissue due to the higher levels of cathepsins found in the tumour microenvironment. Whilst no studies have investigated the use of LUM015 as an FMP in neuroblastoma, seven pre-clinical studies have investigated the expression of cathepsins in neuroblastoma [17,19,35,37,44,47,49].

In a 2022 analysis of the role of cathepsin L in neuroblastoma chemoresistance, human tissue samples from the R2 Genomics and Visualisation Platform revealed that higher mRNA levels of cathepsin L were associated with significantly poorer survival and advanced tumour stages, though this finding did not reach statistical significance [17]. There was heterogeneity in the expression of cathepsin L across cell lines, with low levels observed in IMR-32 cells and high levels in SK-N-BE cells.

In a further pre-clinical study of cathepsin expression, a functional network analysis highlighted an enriched cathepsin protein network in the SK-N-BE2 secretome, including CTSL1, CTSD, CTSB, CTSC, CTSA, and CTSL2 [19]. This contributed to an increased chemoresistance to doxorubicin in MYCN-amplified neuroblastoma cell lines compared with non-MYCN amplified lines.

In a 2008 study focusing on the role of cathepsin D (CTSD), this was shown to also confer protection to neuroblastoma cells against doxorubicin-induced apoptosis [35]. While Tet21N cells transfected with the CTSD gene show increased viability after short-term doxorubicin treatment, silencing of the same gene increases sensitivity to doxorubicin. This finding supports the role of cathepsins in chemoresistance in human neuroblastoma cells with MYCN amplification.

Three further studies looked at cathepsin expression in SH-SY5Y neuroblastoma cell lines, with one suggesting that higher levels of CTSD expression increased median survival in a non-statistically significant manner [37,44,47]. A total of 75% of patients with stage 4 neuroblastoma had low expression of CTSD in their tumour tissue. This correlation was further evaluated in further in vitro studies where SH-SY5Y cells were transfected to overexpress CTSD, which was associated with the reduced proliferative potential of clones.

In a 2024 study by the same group, CTSD expression was shown to differentially effect cell growth in adherent and suspension conditions, with the overexpression of CTSD being advantageous for growth in suspension and CTSD knockdown being favoured for adherent growth [49]. This suggests that CTSD expression can be modified to confer a survival advantage during the growth and spread of neuroblastoma cells.

#### 3.2.2. Studies Involving Probes/Targets in Phase 3 Trials

FMPs in phase 3 clinical trials include Surgimab-101, which targets the carcinoembryonic antigen (CEA), and folate fluorescein isothiocyanate (EC17), targeting the folate receptor. None have been investigated in neuroblastoma to date.

#### 3.2.3. Studies Involving Probes/Targets in Phase 2 Trials

##### Epidermal Growth Factor Receptor (EGFR)

EGFR is a transmembrane protein implicated in multiple cancers, with four FMPs currently undergoing clinical trials (QRHKPRE-Cy5, Panitumumab-IRDye800CW, Nimotuzumab-IRDye800CW, and Cetuximab-IRDye800CW). Two studies have investigated the expression of EGFR in neuroblastoma [21,46].

In an analysis of neuroblastoma tissue samples from 25 children, 81% of the tumours expressed EGFR, and it was shown to be localised both on the cell membrane and in the cytoplasm [46]. The expression rate was higher in neuroblastoma with poorer prognosis. A variable level of expression was found in neuroblastoma cell lines, although this was higher than the control human fibroblast lung cell line (HFL1).

Another study of 106 tumour sections also revealed that nearly all tumours expressed EGFR, with high expression levels being observed in 78% of cases [21]. They also found that polymorphisms in EGFR were infrequent in neuroblastoma tissue, suggesting that EGFR might be a common and conserved target in neuroblastoma.

##### Vascular Endothelial Growth Factor Receptor (VEGFR)

VEGFRs are a family of transmembrane tyrosine kinases involved in regulating tumour-induced angiogenesis via signalling with vascular endothelial growth factor (VEGF). VEGFR can be targeted by the FMP Bevacizumab IRDye800CW. Five pre-clinical studies have investigated the expression of VEGF or VEGFR in neuroblastoma [13,25,27,29,34].

Through a gene network analysis of the Cancer Genetics web and STRING databases, VEGF genes were shown to be involved in several pathways underlying the mechanisms of neuroblastoma in the regulation of angiogenesis and metastasis of cancer [13].

The expression of VEGFR in neuroblastoma cell lines and patient-derived tissues was studied as early as 1999 [29]. VEGF mRNA expression has been identified in six neuroblastoma cell lines, with varying levels of expression, as well as in various neuroblastoma tissue samples.

In 2012, Ramani et al. analysed 102 neuroblastoma samples, demonstrating that VEGF-C, VEGF-D, and VEGFR-3 were all expressed in neuroblastoma [34]. VEGFR-3 expression was significantly higher in patients aged less than 18 months at diagnosis and in high-risk neuroblastoma. VEGFR-3 expression was also associated with poorer event-free survival.

##### Integrins

Integrins are a group of transmembrane protein heterodimers formed of α- and β-chains that act as cell adhesion receptors, mediating cell–cell and cell–extracellular matrix adhesion. They can be targeted by the FMPs cRGD-ZW800-1 and cRGDY-PEG-Cy5.5 nanoparticles. We identified three studies which highlighted the expression of integrins in neuroblastoma [18,30,31].

In a study of 45 neuroblastoma specimens, all tumours expressed the β1 integrin chain, while 44 expressed α1 and 42 expressed α3 chains [18]. This expression was shown to correlate with tumour location, with 75% of tumours expressing a4 and β1, located in the mediastinum.

In a later study, it was found that more aggressive neuroblastoma cell lines had lower levels of β1 integrin expression, which correlated with increased cell detachment and migration [30].

Finally, one study investigated the expression of integrin α9 and its correlation with patient survival. The knockdown of integrin α9 (ITGA9) gene in BE(2)-C cells was shown to reduce cell proliferation and impair the ability of cells to engraft and develop metastases in xenograft mouse models, with a reduction of 30% in metastasis formation observed [31].

##### Matrix Metalloproteinases (MMPs)

MMPs are capable of degrading extracellular matrix proteins and regulating the tumour microenvironment. These are targeted by the FMPs BLZ-100 and activate AVB-620 to result in fluorescence. Four studies investigated the expression of these enzymes in neuroblastoma, all of which indicated the expression of MMP-2 in neuroblastoma cell lines and tissue samples [12,14,41,43]. Higher levels of expression were associated with increased invasion and metastasis, indicative of more advanced disease states.

MMP-9 was also shown to be expressed in neuroblastoma tissue, with 94% of specimens testing positive for MMP-9 [41]. The majority of the strong positive rates of expression were seen in specimens from patients with advanced neuroblastoma.

##### Annexin A2

Annexin A2 is a calcium-dependent phospholipid-binding protein involved in cellular transport, membrane domain organisation, signal transduction, and gene regulation. It is targeted by BLZ-100. Two studies investigated the expression of annexin A2 in neuroblastoma [40,45].

In a proteome-wide quantitative comparison of protein expression in human neuroblastoma cell lines with different drug sensitivities, SK-N-BE(1) and SK-N-BE(2), annexin A2 was found to be upregulated by more than 12-fold in the chemo-resistant neuroblastoma cell lines [40]. This was supported by the analysis of 42 patient tissue samples, of which 73.8% expressed annexin A2, with a stronger intensity of staining correlating with an advanced stage of disease.

#### 3.2.4. Studies Involving Probes/Targets in Phase 1 Trials

##### Gastrin-Releasing Peptide Receptor (GRPR)

GRPR is a G-protein coupled receptor involved in regulating the release of gastrointestinal hormones, smooth muscle proliferation and autocrine growth stimulation. It is widely known to be overexpressed in cancer cells and is targeted by the FMP 68Ga-BBN-IRDye800CW. Three studies investigated its expression in neuroblastoma.

In a study of 19 tumour samples, GRPR mRNA was present in all specimens, though this appeared to lack prognostic significance [36]. In another study of 24 tumours, GRPR was expressed to a variable degree, with higher expression in more undifferentiated neuroblastoma [23].

The role of GRPR expression in neuroblastoma was investigated through the silencing of GRPR expression in BE(2) cells. This was shown to be associated with a reduced cellular proliferation and cell size compared with controls [33]. It also reduced anchorage independence, with fewer colonies produced when grown in soft agar.

##### Carbonic Anhydrase IX (CAIX)

CAIX is a tumour-associated cell surface glycoprotein induced by hypoxia and widely implicated in cancer progression, targeted by 111In-DOTA-girentuximab IRDye800Cw. One study investigated the role of CAIX in neuroblastoma [11].

Of 22 patient tissue samples, 7 were found to express CAIX, with no correlation between expression and patient age, neuroblastoma stage or grade, or metastatic dissemination. However, its expression was found to be significantly associated with poorer overall survival.

### 3.3. Clinical Study Quality and Risk of Bias Assessment

Risk of bias was assessed for all included clinical studies (Table 4). On a scale of low, moderate, serious, or critical risk of bias, all four clinical studies were deemed to have a serious risk of bias. Studies were frequently penalised for failing to account for confounding variables, the lack of availability of outcome data for participants, and the lack of blinding of the outcome assessors.

## 4. Discussion

In this review, we systematically identified and analysed 44 papers which describe the use of clinically approved FMPs or the expression of their targets in paediatric neuroblastoma. Most of the studies were pre-clinical and focused on the targets of probes currently in clinical trials for use in other cancers. Commonly studied FMPs include ICG and LUM015, whilst the targets for which the most studies were identified were PARP, VEGFR, and MMPs.

Four clinical studies were included, three of which studied ICG [8,9,20]. The use of ICG in FGS is well-established since it is widely studied, relatively cheap, and generally well-tolerated [50]. However, the included clinical studies demonstrated inconsistencies in the ability of ICG to effectively localise and visualise tumour tissue, demonstrating the potential inadequacy of using this untargeted probe in neuroblastoma. The EPR effect, which contributes to the tumour uptake of ICG, has been shown to be affected by the tumour type and size, the extent of necrosis, the anatomical location, and the presence of vascular mediators [51,52]. It is therefore difficult to predict the intensity and consistency of fluorescence when used in neuroblastoma. Further neuroblastoma-specific research is needed to determine the utility of ICG regarding outcomes such as complete resection, recurrence rates, and survival.

Most of the pre-clinical studies investigated the expression of molecular targets that could potentially be used for FGS. An ideal target would be one that is expressed in a high proportion of neuroblastoma tumour samples, has diffuse expression throughout the entire tumour, is preferably localised on the cell surface of the tumour, remains present following neo-adjuvant therapy, and has shown absent or minimal expression in normal tissues.

The reviewed studies revealed heterogeneity in the expression of targets or FMP activators, such as those investigating cathepsins. Variation in the expression of cathepsins across cell lines was noted in multiple experiments, and there appeared to be an association between certain levels of expression and different stages of disease. Such discrepancies in expression may make cathepsins a poor candidate for the activation of an FMP, since expression is likely to vary between, and perhaps within, tumours.

Many studies suggested that there was an increased expression of targets in *MYCN*-amplified tumours, as was the case for the expression of PARP, and this was associated with increased sensitivity to PARP inhibition [15,24]. There also appeared to be an association of the expression of some targets with more advanced stages of disease or metastasis, suggesting these might not be as useful in earlier disease states that would be more amenable to surgery.

Studies rarely compared the expression of targets to normal tissue. One exception was the work by Makvandi et al., which showed that PARP expression was higher in neuroblastoma when compared with a wide range of healthy tissues [26]. This highlights a need for future pre-clinical studies that compare neuroblastoma tissue expression to other healthy human tissues, particularly those in sites where neuroblastoma is commonly found or to which it metastasises, to ensure adequate tumour-to-background ratios (TBR) are achieved.

### 4.1. Future Implications

FGS is a novel technique that bridges the gap between pre-operative imaging and intraoperative findings, allowing surgeons to differentiate between tumour and healthy tissue in real-time. This is particularly important in neuroblastoma, which can invade and encase surrounding healthy tissues, including blood vessels, nerves, and vital organs. As such, the surgical resection of neuroblastoma tissue is challenging, and complication rates can be high, ranging from 5 to 25% [4,53]. Major complications include vascular injury, nephrectomy, and nerve injury. The disproportionate impact of neuroblastoma on the paediatric population complicates matters further, since surgeons are faced with smaller anatomical structures through which to dissect. FGS permits a clearer visualisation of tumour tissue, maximising the extent of removal whilst protecting nearby healthy structures during surgical resection, which can, in turn, increase survival [54]. Through our work, we demonstrated several potential FMPs and targets that could be used for FGS in neuroblastoma and warrant further pre-clinical validation. The translation of promising FMPs for clinical use in FGS has the potential to arm surgeons with the ability to better visualise tumour tissue in real-time to guide precise and targeted resection with better prognostic outcomes for patients.

### 4.2. Limitations

This systematic review was conducted in a non-biassed manner with papers identified via a comprehensive literature search. To guide the assessment of studies and the strength of the evidence, we assessed clinical study quality using validated tools; however, this was not possible for pre-clinical studies due to the lack of an appropriate risk of bias tool. A key limitation is that most of these findings remain hypothetical, and their clinical applicability is uncertain. Further validation in cell lines, tissue samples, and xenograft models is required before these findings can be translated into clinical practice.

## 5. Conclusions

This review highlights the potential for clinically approved FMPs, and those in clinical trials, used in adult cancers to be translated into neuroblastoma FGS. Whilst few of the FMPs themselves have been directly studied in neuroblastoma, several targets have been highlighted as being expressed in this tumour type. Further work is required to compare the expression of these targets to healthy tissues and to determine their persistence at different stages of treatment. This study provides a foundation to guide further work in prioritising FMPs and targets for future pre-clinical validation in neuroblastoma, with the aim of translating these findings into clinical practice.

## Figures and Tables

**Table 1:** Included fluorescent molecular probes (FMPs).

Probe	Type of FMP	Target/Activator	FDA Approval/Clinical Trial Status	Trial Number	Cancer Studied In
ICG *	Non-specific dye	-	Approved	-	Gastrointestinal cancers, hepatobiliary cancer, breast cancer, gynaecological cancers, head and neck cancer, lung cancer, paediatric cancers (including neuroblastoma)
Methylene blue	Non-specific	-	Approved	-	Breast cancer, neuroendocrine tumours, colorectal cancer
Fluoroscein	Non-specific	-	Approved	-	CNS cancer
5-aminolevulinic acid *	Non-specific	-	Approved	-	Head and neck cancer, CNS cancer
Pafolacianine (OTL38)	Targeted	Folate receptor alpha	Approved	-	Ovarian cancer, lung cancer
Lumicell (LUM015) *	Activatable	Cathepsin	Approved	-	Breast cancer, sarcoma
Surgimab-101	Targeted	CEA	Phase III	NCT03659448	Colorectal cancer
Folate fluorescein isothiocyanate (EC17)	Targeted	Folate receptor	Phase III	2017-004557-17, NCT01511055	Ovarian cancer, breast cancer, renal cell carcinoma, lung cancer
Panitumumab-IRDye800CW *	Targeted	EGFR	Phase II	NCT04511078, NCT03384238	Head and neck cancer, pancreatic cancer
Bevacizumab IRDye800CW *	Targeted	VEGFR	Phase II	NCT05359874	Breast cancer, thyroid cancer, rectal cancer, sarcoma, cholangiocarcinoma, oesophageal cancer, pancreatic cancer, head and neck
cRGD-ZW800-1 *	Targeted	Integrins	Phase II	NCT05518071, NCT04191460, NCT05752149	Pancreas adenocarcinoma, head and neck cancer, laryngeal cancer
BLZ-100 *	Targeted	Annexin A2, MMP2	Phase II	NCT02234297, NCT02097875, NCT03579602	Glioma, skin neoplasms, oral cavity SCC, paediatric CNS tumours
Onconano medicine	Targeted	ONM-100	Phase II	NCT03735680	Breast cancer, head and neck SCC, colorectal, prostate, ovarian, urothelial, non-small cell lung cancer
AVB-620 *	Activatable	MMP2 and 9	Phase II	NCT03113825	Breast cancer
EMI-137	Targeted	c-MET	Phase II	NCT03360461, NCT03470259, NCT03205501	Colorectal cancer, Thyroid cancer, oesophageal cancer
Cetuximab-IRDye800CW *	Targeted	EGFR	Phase I-II	NCT02855086, NCT02736578 (terminated)	Glioma, pancreatic cancer
cRGDY-PEG-Cy5.5 nanoparticles *	Targeted	Integrins	Phase I–II	NCT02106598	Head and neck cancer, melanoma
PARPi-FL *	Targeted	PARP	Phase I–II	NCT03085147, NCT03631017	Oral SCC, head and neck cancer
VST-1001 (dilute fluorescein)	Non-specific	-	Phase I–II	NCT02294565	Breast cancer
[111In]In-DOTA-Labetuzumab-IRDye800CW	Targeted	CEA	Phase I–II	NCT03699332	Colorectal cancer
Nimotuzumab-IRDye800CW *	Targeted	EGFR	Phase I–II	NCT04459065	Lung cancer
VST-1001 (dilute fluorescein)	Non-specific	-	Phase I–II	NCT02294565	Breast cancer
[111In]In-DOTA-Labetuzumab-IRDye800CW	Targeted	CEA	Phase I–II	NCT03699332	Colorectal cancer
VGT-309 *	Activatable	Cathepsin	Phase I	NCT05400226, NCT06145048, NCT06034197	Lung cancer, colorectal cancer
111In-DOTA-girentuximab IRDye800Cw *	Targeted	CAIX	Phase I	NCT02497599	Renal cell carcinoma
FluoAB	Non-specific	-	Phase I	NCT05394246	Liver cancer
68Ga-BBN-IRDye800CW *	Targeted	GRPR	Phase I	NCT03407781, NCT02910804	Glioma, glioblastoma
QRHKPRE-Cy5 *	Targeted	EGFR	Phase I	NCT02574858	N/A–healthy adults
RD0Cy7 fluorophore	Targeted	ITGA6	Phase I	NCT06204835	Hepatocellular carcinoma

* Indicates FMPs or FMPs with targets amenable for use in neuroblastoma included in the results of this review.

**Table 2:** Summary of included studies.

Author	Year	Study Design	Type of Sample	Fluorescent Dye/Probe	Target	Reference
Papers Investigating Probe Accumulation Within Tumour Tissue						
Abdelhafeez et al.	2021	Clinical	Patients	ICG	Untargeted	[8]
Abdelhafeez et al.	2023	Clinical	Patients	ICG	Untargeted	[9]
Ak et al.	2015	Preclinical	Cell lines	ICG	Untargeted	[10]
Di Giulio et al.	2021	Preclinical	Cell lines	PARPi	PARP	[16]
Harris et al.	2023	Clinical	Patients	ICG	Untargeted	[20]
King et al.	2020	Preclinical	Cell lines	PARPi	PARP	[24]
Keller et al.	2023	Preclinical	Genomics of Drug Sensitivity in Cancer database; cell lines	PARPi	PARP	[22]
Lai et al.	2024	Preclinical	Cell lines	PARPi	PARP	[48]
McNeil et al.	2013	Preclinical	Cell lines	PARPi	PARP	[28]
Norris et al.	2014	Preclinical	Cell lines, xenograft models	PARPi	PARP	[32]
Takagi et al.	2017	Preclinical	Tissue samples, cell lines, xenograft models	PARPi	PARP	[38]
Takagi et al.	2022	Clinical	Patients	PARPi	PARP	[39]
Watanabe et al.	2022	Preclinical	Cell lines	5-ALA	Untargeted	[42]
Papers Investigating Target Expression Within Tumour Samples						
Ameis et al.	2015	Preclinical	Tissue samples, cell lines	111In-DOTA-girentuximab IRDye800Cw	CAIX	[11]
Ara et al.	1998	Preclinical	Tissue samples	BLZ-100, AVB-620	MMP-2	[12]
Ashok et al.	2021	Preclinical	Cancer genetics web database	Bevacizumab IRDye800CW	VEGF/VEGFR	[13]
Bjornland et al.	2001	Preclinical	Cell lines	BLZ-100, AVB-620	MMP-2	[14]
Colicchia et al.	2017	Preclinical	Cell lines	PARPi-FL	PARP	[15]
Du et al.	2022	Preclinical	Cell lines, xenograft models	Lumicell (LUM015), VGT-309	Cathepsin	[17]
Favrot et al.	1991	Preclinical	Tissue samples	cRGD-ZW800-1, cRGDY-PEG-Cy5.5 nanoparticles, RD0Cy7 fluorophore	Integrins	[18]
Gallwitz et al.	2024	Preclinical	Cell lines	Lumicell (LUM015), VGT-309	Cathepsin	[47]
Gangoda et al.	2015	Preclinical	Cell lines	Lumicell (LUM015), VGT-309	Cathepsin	[19]
Izycka-Swieszewska et al.	2010	Preclinical	Tissue samples	Panitumumab-IRDye800CW, Cetuximab-IRDye800CW, Nimotuzumab-IRDye800CW, QRHKPRE-Cy5	EGFR	[21]
Kim et al.	2002	Preclinical	Cell lines, tissue samples	68Ga-BBN-IRDye800CW	GRP/GRPR	[23]
Langer et al.	2000	Preclinical	Cell lines, tissue samples	Bevacizumab IRDye800CW	VEGF/VEGFR	[25]
Makvandi et al.	2019	Preclinical	Tissue samples	PARPi-FL	PARP-1	[26]
Marcus et al.	2005	Preclinical	Cell lines, xenograft models, tissue samples	Bevacizumab IRDye800CW	VEGF/VEGFR	[27]
Meister et al.	1999	Preclinical	Cell lines, tissue samples	Bevacizumab IRDye800CW	VEGF/VEGFR	[29]
Meyer et al.	2004	Preclinical	Cell lines	cRGD-ZW800-1, cRGDY-PEG-Cy5.5 nanoparticles, RD0Cy7 fluorophore	Integrins	[30]
Navarro et al.	2022	Preclinical	Cell lines, xenograft models	cRGD-ZW800-1, cRGDY-PEG-Cy5.5 nanoparticles, RD0Cy7 fluorophore	Integrins	[31]
Qiao et al.	2008	Preclinical	Cell lines, xenograft models	68Ga-BBN-IRDye800CW	GRPR	[33]
Ramani et al.	2012	Preclinical	Tissue samples	Bevacizumab IRDye800CW	VEGFR	[34]
Sagulenko et al.	2008	Preclinical	Cell lines	Lumicell (LUM015), VGT-309	Cathepsin	[35]
Sebesta et al.	2001	Preclinical	Tissue samples	68Ga-BBN-IRDye800CW	GRP/GRPR	[36]
Secomandi et al.	2022	Preclinical	Cell lines	Lumicell (LUM015), VGT-309	Cathepsin D	[37]
Secomandi et al.	2024	Preclinical	Cell lines	Lumicell (LUM015), VGT-309	Cathepsin D	[49]
Wang et al.	2017	Preclinical	Cell lines	BLZ-100	Annexin A2	[40]
Wang et al.	2020	Preclinical	Tissue samples	BLZ-100, AVB-620	MMP2	[41]
Yuan et al.	2021	Preclinical	Cell lines, tissue samples	BLZ-100, AVB-620	MMP-2	[43]
Zhang et al.	2017	Preclinical	Cell lines, tissue samples, xenograft mouse models	Lumicell (LUM015), VGT-309	Cathepsin	[44]
Zhang et al.	2023	Preclinical	Patient venous blood samples, cell lines	BLZ-100	Annexin A2	[45]
Zheng et al.	2016	Preclinical	Cell lines, tissue samples	Panitumumab-IRDye800CW, Cetuximab-IRDye800CW, Nimotuzumab-IRDye800CW, QRHKPRE-Cy5	EGFR	[46]

**Table 3:** Summary of included human clinical studies.

First Author and Year	Study Design	Probe Investigated	Neuroblastoma Patient Cohort	Age Range (Years)	Reference
Abdelhafeez, 2021	Retrospective cohort study	ICG	6	<1–21	[8]
Abdelhafeez, 2023	Phase I trial	ICG	1	<1–23	[9]
Harris, 2023	Phase I trial	ICG	3	<1–4.1	[20]
Takagi, 2022	Phase I trial	Olaparib (PARPi)	6	3–18	[39]

**Table 4:** Risk of bias assessment of clinical studies.

First Author and Year	Study Design	ROB Assessment Tool	ROB Assessment Grading	Reference
Abdelhafeez, 2021	Retrospective cohort study	ROBINS-I	Serious	[8]
Abdelhafeez, 2023	Single-centre, open-label, nonrandomised, prospective clinical trial	ROBINS-I	Serious	[9]
Harris, 2023	Single-centre, open-label, nonrandomised, prospective clinical trial	ROBINS-I	Serious	[20]
Takagi, 2022	Multi-centre, open-label, nonrandomised, prospective clinical trial	ROBINS-I	Serious	[39]

## Supplementary Materials

The following supporting information can be downloaded at: https://www.mdpi.com/article/10.3390/children12050550/s1, Supplementary Materials S1 (search strategy) and Supplementary Materials S2 (abstract decision tree).

## Abbreviations

The following abbreviations are used in this manuscript:

FGS	Fluorescence-guided surgery
FMP	Fluorescent molecular probes
ICG	Indocyanine green
5-ALA	5-aminolevulinic acid
PARP	Poly-ADP ribose polymerase
VEGFR	Vascular endothelial growth factor receptor
EGFR	Endothelial growth factor receptor
EPR	Enhanced permeability and retention
CTS	Cathepsin
ITG	Integrin
GRPR	Gastrin-releasing peptide receptor
CAIX	Carbonic anhydrase IX
MMP	Matrix metalloproteinase
