# Supplementary material for: Identifying Molecular Probes for Fluorescence-Guided Surgery in Neuroblastoma: A Systematic Review
Source: Children (Basel). 2025 Apr 24;12(5):550. doi: 10.3390/children12050550 (PMC12110316; doi:10.3390/children12050550)
Supplement: Supplementary file 1 [file children-12-00550-s001.zip › children-3566798-supplementary.pdf]

Identifying Molecular Probes for Fluorescence-Guided Surgery in Neuroblastoma: A Systematic Review

Megan Hennessy, Jonathan J. Neville, Laura Privitera, Adam Sedgwick, John Anderson and Stefano Giuliani

Contents

Supplementary materials S1. Search strategy

Supplementary materials S2. Abstract decision tree

S1: Search strategy

Search #	Search terms
1	[neuroblastoma OR neuroblastomas OR neuroblast* OR ganglioneuroblastoma OR ganglioneuroblastomas OR ganglioneuroblast* OR neuroepithelioma OR neuroepitheliomas OR neuroepitheliom* OR esthesioneuroblastoma OR esthesioneuroblastomas OR esthesioneuroblastom* OR schwannian]
AND	
2	[indocyanine green OR ICG] OR [methylene blue] OR [fluorescein] OR [5-aminolevulinic acid OR 5-ALA] OR [surgimab-101 OR SGM-101] OR [cetuximab-IRDye800CW OR EGFR] OR [panitumumab-IRDye800CW OR EGFR] OR [Bevacizumab-IRDye800CW OR VEGFR] OR [cRGD-ZW800-1 OR integrins] OR [PLSWT7-DMI OR CD44V6] OR [BLZ-100 OR Annexin A2 OR MMP2] OR [folate fluorescein isothiocyanate OR EC17 OR folate receptor] OR [pafolacianine OR OTL38 OR folate receptor alpha] OR [cRGDY-PEG-Cy5.5 nanoparticles OR integrins] OR [onconano medicine OR ONM-100] OR [lumicell OR LUM015 OR cathepsin] OR [AVB-620 OR MMP] OR [111In-DOTA-girentuximab-IRDye800CW OR CAIX] OR [68Ga-IRDye800CW-BBN OR GRPR] OR [PARPi-FL OR PARP] OR [QRHKPRE-Cy5-KSPNPRE-IRDye800CW OR EGFR] OR [Anti-CD47-FITC OR anti-CD47–Qdot625 OR CD47] OR [EMI-137 OR c-MET] OR [EP-HMRG OR DPP-IV] OR [GB119 OR cathepsin] OR [6qc-NIR OR cathepsin] OR [VST-1001 fluorescein] OR [VGT-309 OR cathepsin] OR [111In-DOTA-labetuzumab-IRDye800CW OR CEA] OR [FluoAB] OR [RD0Cy7 fluorophore OR integrin OR ITGA6]

S2: Abstract decision tree

Step	Criteria	Decision
1	Is it a primary clinical or pre-clinical study?	Yes – move to step 2<br>No – exclude
2	Is it a primary study of paediatric neuroblastoma?	Yes – move to step 3<br>No – exclude
3	Does the study consider one of the following?<br>1. Testing an FMP in patients with neuroblastoma<br>2. Investigating selective accumulation of a FMP in neuroblastoma tissue<br>3. Investigating expression of an FMP target in neuroblastoma tissue	Yes – move to step 4<br>No – exclude
4	Confirm steps 1-3, is the study a primary clinical or pre-clinical study in paediatric neuroblastoma and does it report an outcome of interest?	Yes – include<br>No – revisit 1-3
